# Magnetic compression anastomosis with atypical anastomosis for anastomotic stenosis of the sigmoid colon: a case report

**DOI:** 10.1186/s40792-020-00826-9

**Published:** 2020-03-30

**Authors:** Teppei Kamada, Hironori Ohdaira, Sojun Hoshimoto, Satoshi Narihiro, Norihiko Suzuki, Rui Marukuchi, Hideyuki Takeuchi, Masashi Yoshida, Eigoro Yamanouchi, Yutaka Suzuki

**Affiliations:** 1 Department of Surgery, International University of Health and Welfare Hospital, 537-3, Iguchi, Nasushiobara, Tochigi 329-2763 Japan; 2 Department of Radiology, International University of Health and Welfare Hospital, 537-3, Iguchi, Nasushiobara, Tochigi 329-2763 Japan

**Keywords:** Magnetic compression anastomosis, Side-to-side anastomosis

## Abstract

**Background:**

Magnetic compression anastomosis (MCA) is mainly applied in the gastrointestinal and biliary tracts through a nonsurgical procedure that can create an anastomosis similar to that obtained through surgery.

Magnets usually adsorb in the end-to-end direction (end-to-end anastomosis), exert a strong magnetic force and create an anastomosis according to the size of the magnets.

Regular endoscopic dilation is required to prevent restenosis when the anastomotic size is small.

We report a case in which MCA was successfully used to treat anastomotic stenosis of the sigmoid colon; the magnets adsorbed in the side-to-side direction rather than the end-to-end direction and generated a wide anastomosis in a short time that did not require endoscopic dilation.

**Case presentation:**

An 81-year-old woman was admitted to our hospital to treat anastomotic stenosis of the sigmoid colon for closure of transverse colostomy. Two years prior, the Hartmann operation and drainage were performed at other hospitals due to perforated diverticulitis of the sigmoid colon. Obstruction of the sigmoid colostomy occurred, and a transverse colostomy was performed. One year after the first surgery, high anterior resection was performed, but anastomotic stenosis occurred, causing obstruction. MCA was planned because the patient had a history of multiple operations and was expected to have strong adhesions postoperatively. MCA was safely performed, but two magnets were accidently adsorbed in the side-to-side direction. The magnet position could not be changed. The two magnets were expected to move and adsorb in an end-to-end direction naturally due to bowel movements. The magnets that adsorbed in the side-to-side direction dropped from the anus 5 days after treatment, and the anastomosis was observed by colonoscopy. Three ileus tubes were placed from the transverse colostomy beyond the anastomosis to prevent restenosis. Colonoscopy showed that the anastomosis diameter was wider than expected at 14 days after treatment, and endoscopic dilation was not necessary. No complications were observed in this patient’s postoperative course. Finally, closure of the patient’s colostomy was successfully performed.

**Conclusions:**

MCA with side-to-side anastomosis generated a wide anastomosis in a short time.

## Background

Magnetic compression anastomosis (MCA) is a novel interventional method that creates an anastomosis using two rare earth magnets in the targeted segments of the gastrointestinal tract.

MCA is mainly applied in the gastrointestinal and biliary tracts and is a nonsurgical procedure that can create an anastomosis similar to that obtained by surgery.

MCA can be applied even for patients with ascites caused by peritoneal dissemination or patients who are unable to undergo general anesthesia [1].

It has been reported that MCA has few complications, but anastomotic leakage, stenosis, injury to other organs, and deviation and aberration of the magnets are sometimes reported [2].

**Fig. 1:**
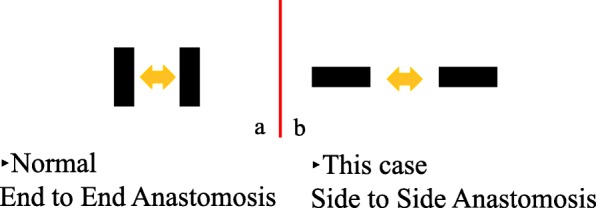
Scheme of adsorption of the two magnets. **a** Normal case: adsorption in the end-to-end direction. **b** This case: adsorption in the side-to-side direction

Magnets usually adsorb into the end-to-end direction (end-to-end anastomosis), resulting in a strong magnetic force and the creation of an anastomosis according to the size of the magnet (Fig. 1).

Endoscopic dilation is required regularly to prevent restenosis when the anastomosis size is small.

We report a case in which MCA was used successfully to treat anastomotic stenosis of the sigmoid colon; the magnets adsorbed in the side-to-side direction and generated a wide anastomosis in a short time, and the patient did not require endoscopic dilation.

## Case presentation

An 81-year-old woman was admitted to our hospital to treat anastomotic stenosis of the sigmoid colon for closure of a transverse colostomy. Two years ago, the Hartmann operation and drainage were performed at another hospital due to perforated diverticulitis of the sigmoid colon.

Obstruction of the sigmoid colostomy occurred, and a transverse colostomy was performed. One year after the first surgery, high anterior resection was performed, but anastomotic stenosis causing obstruction occurred.

**Fig. 2:**
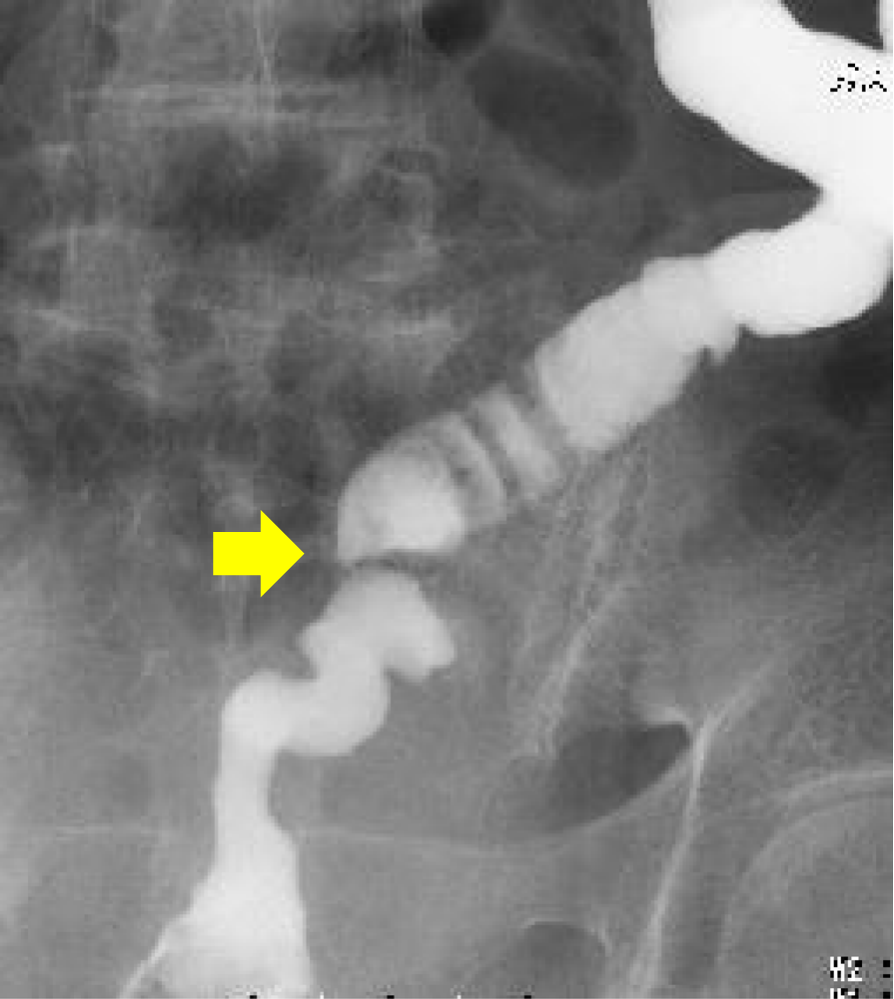
Severe stenosis of anastomosis of the sigmoid colon before treatment (arrows)

Endoscopic dilation was impossible because the guidewire could not be passed (Fig. 2).

MCA was planned due to the patient’s history of multiple operations and the expectation of the patient would develop severe intraabdominal adhesions.

The protocol used for MCA was approved by the Ethics Committee for Biomedical Research of the International University of Health and Welfare Hospital, and the patient was provided an informed consent (Approval No. 13-B-90).

**Fig. 3:**
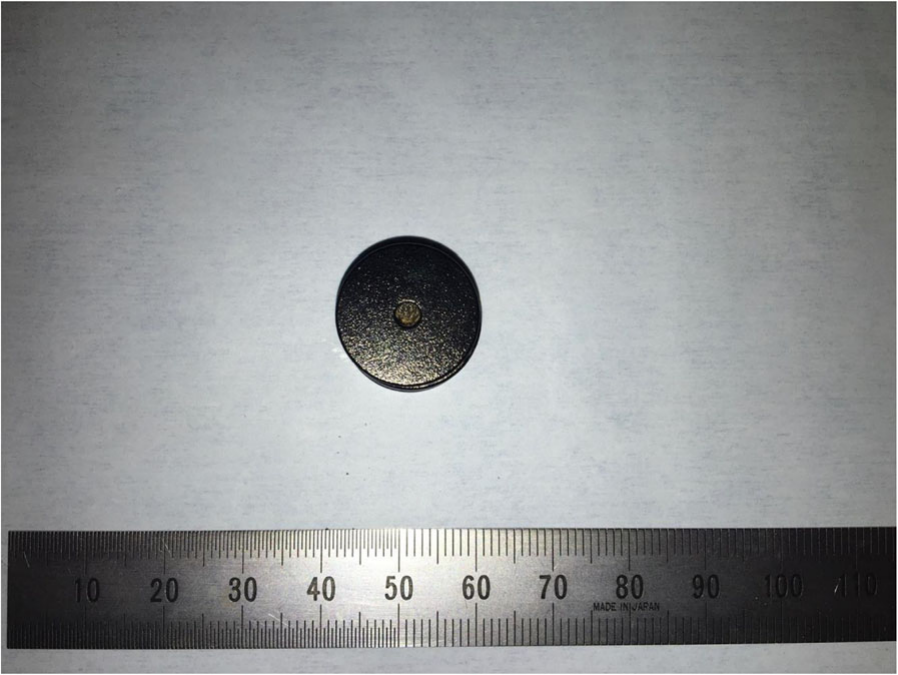
The magnets used for MCA (parent magnet = daughter magnet)

Both the parent magnet (diameter, 17.5 mm; thickness, 5 mm) and daughter magnet (diameter, 17.5 mm; thickness, 5 mm) were cylinders (Fig. 3).

The magnets were constructed of samarium-cobalt.

The daughter magnet was placed on the oral side of the transverse colostomy using an ileus tube, and the parent magnet was placed on the anal side by colonoscopy.

**Fig. 4:**
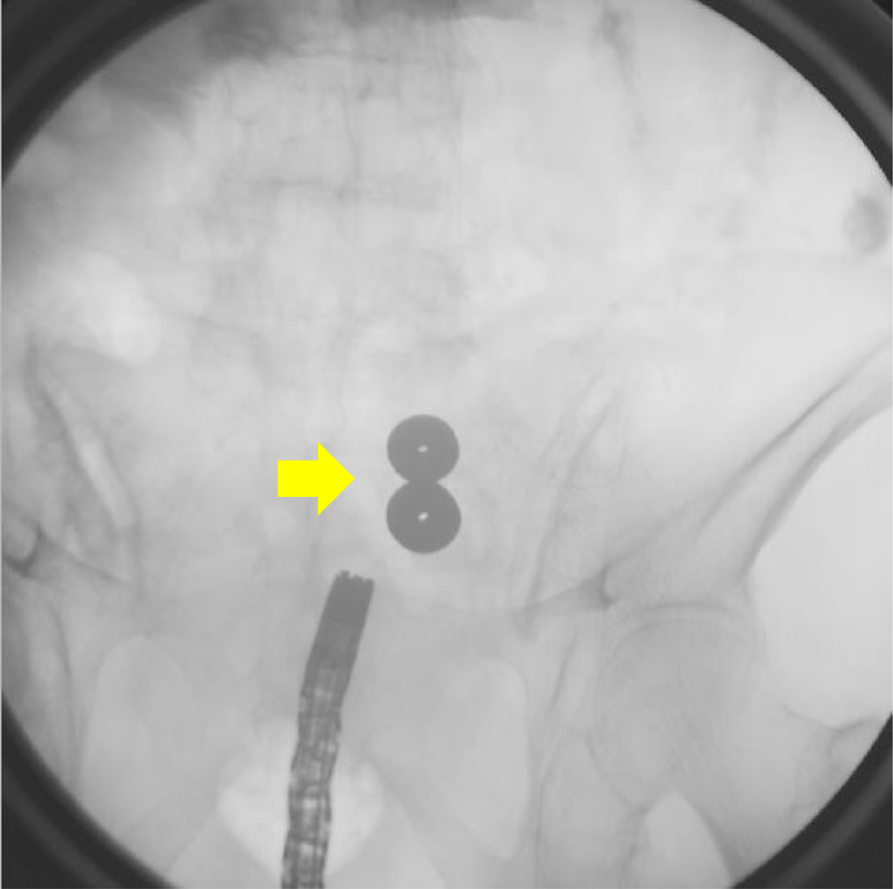
Adsorption in the side-to-side direction during treatment (arrows)

The two magnets were accidentally adsorbed in the side-to-side direction (Fig. 4).

The magnet position could not be changed. The two magnets were expected to move and adsorb in the end-to-end direction naturally die to bowel movements. Furthermore, side-to-side anastomosis has a small adsorption area, so the risk of injury to other organs is considered to be lower than it would be for end-to-end anastomosis if the situation will not change. The magnet position was confirmed on X-ray each day, but adsorption in the side-to-side direction continued.

**Fig. 5:**
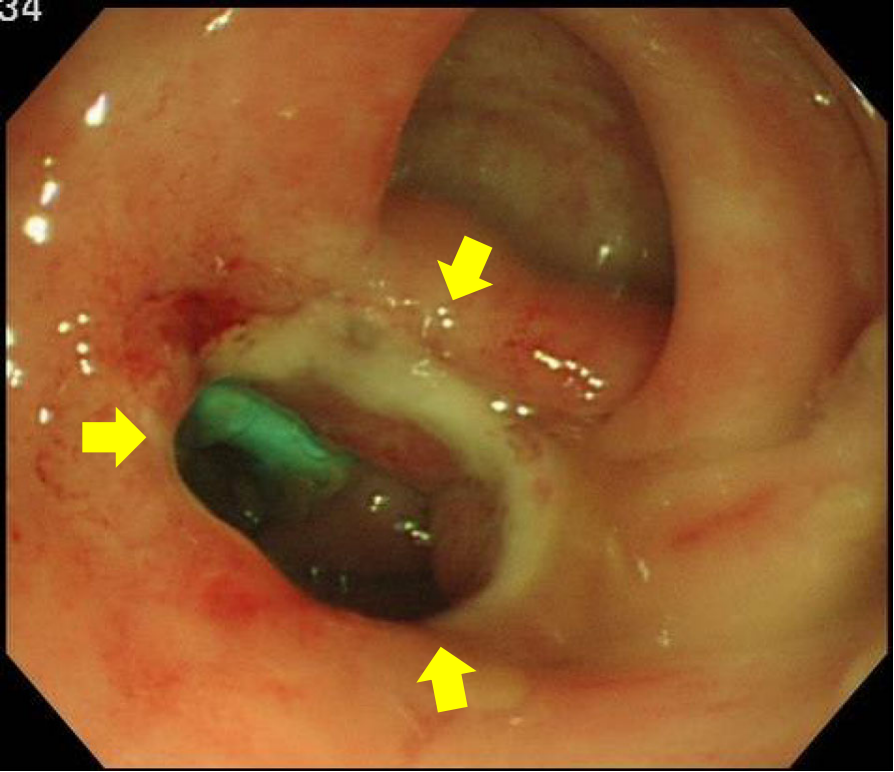
Anastomosis observed by colonoscopy immediately after the magnets were dropped (arrows)

The magnets that had adsorbed in the side-to-side direction dropped from the anus 5 days after treatment, and anastomosis was confirmed by colonoscopy (Fig. 5).

**Fig. 6:**
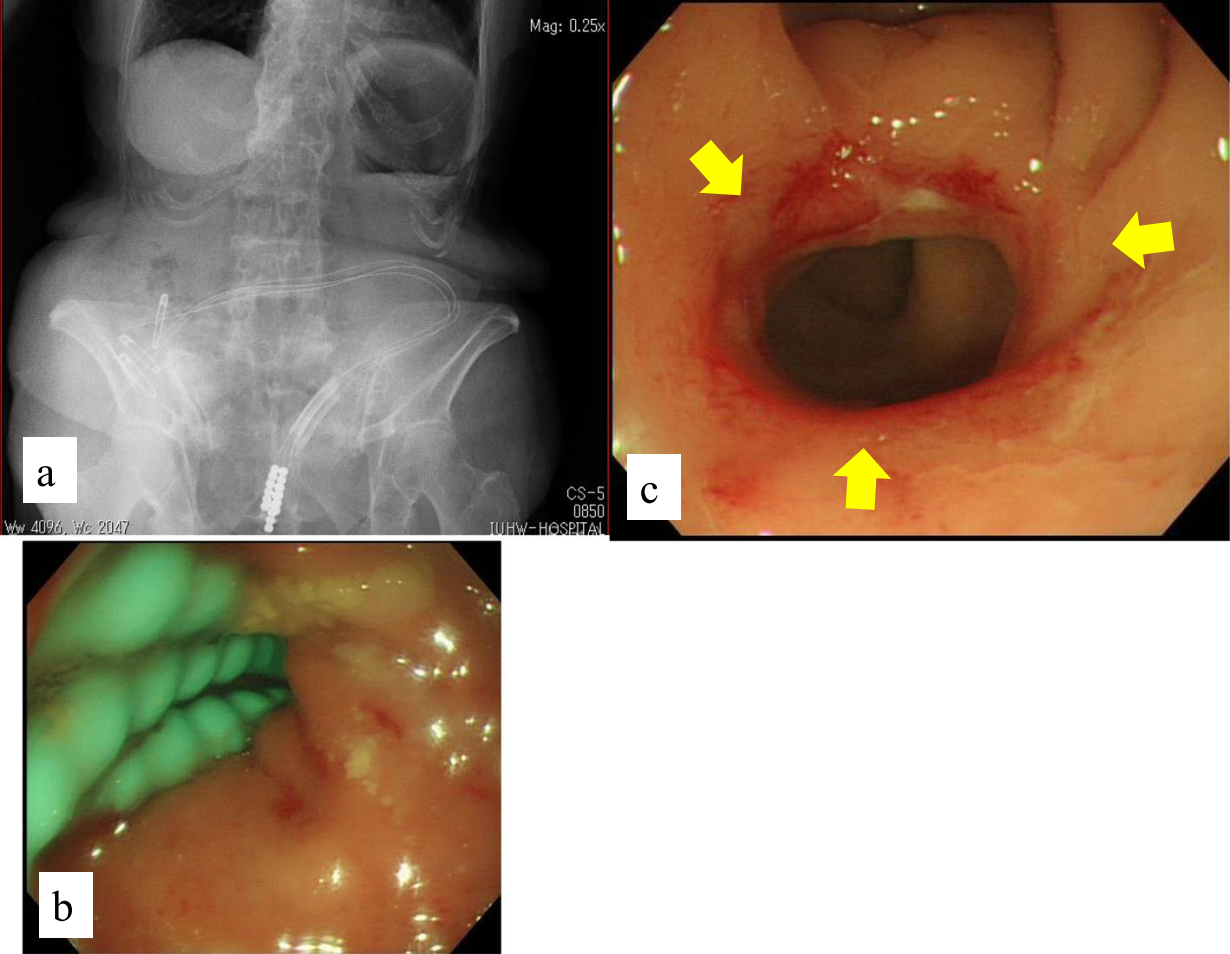
**a**, **b** Three ileus tubes were placed from the transverse colostomy beyond the anastomosis to prevent restenosis. **c** Anastomosis observed by colonoscopy 14 days after treatment (arrows)

An ileus tube was placed from the transverse colostomy beyond the anastomosis to prevent restenosis. Two more ileus tubes were placed every 4 days, and three ileus tubes were finally placed beyond the anastomosis (Fig. 6).

Endoscopic dilation was planned 14 days after treatment, but colonoscopy showed that the anastomosis diameter was wider than expected, and endoscopic dilation was therefore not necessary. No complications were observed in this patient’s postoperative course.

Finally, closure of the patient’s colostomy was successfully performed.

## Discussion

MCA is a safe and unique technique for the reconstruction of entericoenteric, biliobiliary, or bilioenteric anastomosis complications without surgical intervention.

Yamanouchi et al. [3, 4] reported the first use of MCA in the 1990s, and MCA has been performed to treat complications such as obstruction of esophagojejunostomy after total gastrectomy or ileus for patients unsuitable for general anesthesia, stenosis of the bile duct after intraoperative bile duct injury, and stricture of choledochojejunostomy after living donor transplantation [5–7].

In 2019, Watanabe et al. [8] reported the feasibility of hybrid fluorescent magnetic gastrojejunostomy in a porcine model and human cadavers, and Slater et al. [9] reported a minimally invasive approach for anastomosis in esophageal atresia using magnets.

The two magnets absorbed in the side-to-side direction (side-to-side anastomosis) after MCA for refractory anastomotic stenosis of the sigmoid colon in this case.

Magnets usually adsorb into the end-to-end direction (end-to-end anastomosis), resulting in a strong magnetic force and the creation of an anastomosis according to the size of the magnet.

Our search of the literature revealed that all reported cases of MCA led to end-to-end anastomosis. The two magnets were expected to move and adsorb in the end-to-end direction naturally by bowel movements, so the patient was followed up by X-ray without reintervention.

The magnets that adsorbed in the side-to-side direction dropped from the anus 5 days after treatment, and anastomosis was complete. Side-to-side anastomosis was expected to cause restenosis and require frequent endoscopic dilation because the magnetic adsorption area was so small.

However, colonoscopy showed that the anastomosis diameter was wider than expected, and endoscopic dilation was not necessary.

One of the causes of this outcome is that there was not a wide space where the 17.5 mm magnet was located in the end-to-end direction (end-to-end anastomosis) in the sigmoid colon due to severe stenosis.

The possible cause of the larger anastomosis was that the two magnets moved bit by bit around the adsorption area, and there was no residual mucosa because the two magnets were unstable due to the small adsorption area.

In this case, the period to complete anastomosis was shorter (5 days) than typically reported.

At our institution, the average period to complete anastomosis was 13 days (10–19 days), and end-to-end anastomosis was performed in all cases using the same magnets (17.5 mm, samarium-cobalt magnet) (6 cases: 2 cases of esophagus-jejunum, 2 cases of jejunum-jejunum, and 2 cases of jejunum-descending colon).

Considering that side-to-side anastomosis has a smaller adsorption area than end-to-end anastomosis, it is likely that the period to complete anastomosis depends on the size of the adsorption area.

The risk of adverse events in side-to-side anastomosis is unknown, and further studies in more patients are necessary to resolve this issue. In summary, these results suggest that it might not be necessary to change the magnet position with retreatment, and side-to-side anastomosis may require less time than end-to-end anastomosis, even if the two magnets adsorb in the side-to-side direction involuntarily.

## Conclusions

MCA with side-to-side anastomosis might be capable of creating a sufficient anastomosis in a short time.

## Data Availability

Data sharing is not applicable to this article as no datasets were generated or analyzed during the current study.

## Abbreviations

MCA: Magnetic compression anastomosis
